# Fundus changes in type III membranoproliferative glomerulonephritis: a case report

**DOI:** 10.1186/s12886-018-0738-x

**Published:** 2018-03-06

**Authors:** Masato Takei, Akira Obana, Takenori Inomata, Takao Tanaka, Tina Shiang, Yuan Bae, Tamiko Takemura, Akira Murakami

**Affiliations:** 1 Department of Ophthalmology, Japan Red Cross Medical Center, 4-1-22, Hiroo, Shibuya-ku, Tokyo, Japan; 2 Department of Ophthalmology, Juntendo University Faculty of Medicine, 3-1-3, Hongo, Bunkyo-ku, Tokyo, 113-0033 Japan; 3 Department of Ophthalmology, Seirei Hamamatsu General Hospital, 2-12-12, Sumiyoshi, Naka-ku, Hamamatsu, Shizuoka, Japan; 4 Department of Strategic Operation Management and Improvement, Juntendo University Faculty of Medicine, 3-1-3, Hongo, Bunkyo-ku, Tokyo, Japan; 5 Ebisu Eye Clinic, 1-1-2, Ebisunishi, Shibuya-ku, Tokyo, Japan; 6 Orange Park Medical Center, Jacksonville, FL USA; 7 Department of Pathology, Japan Red Cross Medical Center, 4-1-22, Hiroo, Shibuya-ku, Tokyo, Japan

**Keywords:** Membranoproliferative glomerulonephritis type III, Drusen, Choroidal neovascularization

## Abstract

**Background:**

Membranoproliferative glomerulonephritis (MPGN) is characterized by mesangial cell proliferation and is classified into types I, II and III based on structural changes in the glomerular capillary walls. The drusen-like deposits of MPGN type II have been studied, but the fundus changes in MPGN type III have yet to be clarified. We report a case of MPGN type III with multiple deposits in the retinal pigment epithelium (RPE).

**Case presentation:**

A 40-year-old Japanese woman with MPGN type III developed numerous yellow-white patches in the central macula of both eyes. Optical coherence tomography (OCT) showed deposits between the RPE and Bruch’s membrane. Fluorescein angiography showed choroidal neovascularization (CNV) and OCT confirmed it as type 1 (sub RPE) CNV with fibrin tissue and subretinal fluid in the right eye. After 12 months, the CNV and subretinal fluid resolved spontaneously but the RPE deposits remained in both eyes. Her final visual acuity was 20/20 in the right eye and 20/16 in the left eye.

**Conclusion:**

We report a case of MPGN type III with multiple deposits in the RPE and CNV, suggesting that various fundus changes occur in MPGN type III and careful fundus follow-up is necessary to prevent vision loss.

## Background

Membranoproliferative glomerulonephritis (MPGN) is an uncommon kidney disorder characterized by mesangial cell proliferation with electron dense deposits and structural changes in the glomerular capillary walls. MPGN accounts for 4% of nephrosis in children and 7% nephrosis in adults, with higher prevalence in women [1]. MPGN is clinically classified as either primary (idiopathic) or secondary MPGN [2], and histologically classified as types I, II and III according to the location of electron dense deposits [3]. MPGN type II, also known as a dense deposit disease (DDD), is caused by abnormalities in the alternative complement pathway, resulting in deposition of complement C3 in the glomerular basement membrane. The etiologies of MPGN type III have not been fully elucidated, but are considered to be different from type II [4]. Immune complex deposits were observed under glomerular endothelial cells in type I and under both glomerular endothelial cells and epithelial cells in type III. The disease demographics also vary between MPGN subtypes. Type I is found in adolescents and people in their 40s. Type II is commonly found in teenagers, many of which have highly active disease. Type III can occur at any age.

In previous studies, fundus abnormalities were mostly reported in MPGN type II, described as drusen-like deposits in the basement membrane of Bruch’s membrane and associated with choroidal neovascularization (CNV) and idiopathic central serous chorioretinopathy [5–7]. In MPGN type I, drusen-like deposits were also reported but the extent varied, ranging from no fundus abnormalities to extensive drusen-like deposits [8].

To our knowledge, there have been few reports describing fundus changes associated with MPGN type III [4]. This case demonstrates MPGN type III with multiple retinal deposits and describes the 1 year follow-up findings.

## Case presentation

**Fig. 1:**
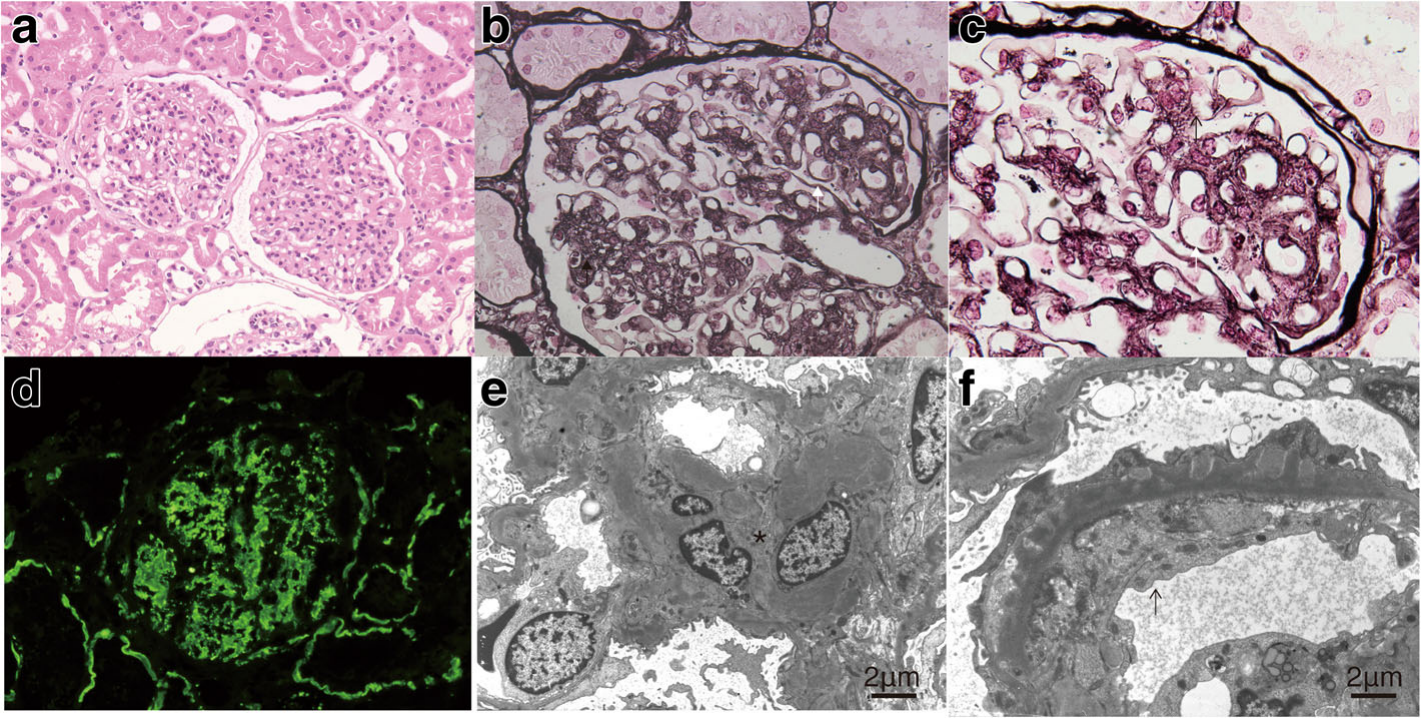
Renal biopsy. **a** hematoxylin and eosin (H&E) staining shows glomerular morphology noting increased cellularity and mesangial expansion (**b** and **c**). High magnification of periodic acid methenamine silver (PAM) staining shows the double loop sign (**b** and **c**, **white arrow**) and bubbling appearance (**e**, **black arrow**). **d** Fluorescence immunostaining shows granular deposition of complement C3 on the glomerulocapillary wall. **e** Electron microscopy shows mesangial cell proliferation and expansion (**e**, **star**) and electron dense deposits (subepithelial immune deposits) on the glomerular basement membrane intercalated into the mesangial cells (**f**, **black arrow**)

A 40-year-old Japanese woman complained of bilateral metamorphopsia. She had bilateral retinochoroiditis with unknown origin and was referred to the Japanese Red Cross Medical Center, Department of Ophthalmology, Tokyo, Japan in December 2016. She had a history of proteinuria at age 8 and was diagnosed with MPGN type III by renal biopsy in 2011. The laboratory tests showed proteinuria (urinary protein 0.60 g/gCre) and hematuria. Urinalysis showed RBC count of 10-19/high power field (HPF) and WBC count of 1-4/HPF. Hemoglobin (13.4 g/dL), total protein (7.2 g/dL) and albumin (4.2 g/dL) were in normal ranges. Hepatitis B surface antigen and hepatitis C virus antibodies were negative. Immunoglobulin levels were in normal ranges (IgG 1290 mg/dL, IgA 367 mg/dL, IgM 129 mg/dL). Anti-nuclear antibody and anti-neutrophil cytoplasmic antibody were negative. Renal function was normal (blood urea nitrogen 20.0 mg/dL, serum creatinine 0.67 mg/dL, estimated glomerular filtration rate 80.1 mL/min). C3 levels (53 mg/dL) was low, and CH50 (44 U/m) and C4 (22.2 mg/dL) were normal. Renal biopsy showed a lobular appearance of the glomerular tuft with increased glomerular cellularity on hematoxylin and eosin (H&E) staining (Fig. 1a). Periodic acid methenamine silver (PAM) staining showed the double loop sign representing interposition of mesangial cell elements with new glomerular basement membrane synthesis (Fig. 1b and c**, white arrow**) and has a bubbling appearance (Fig. 1c **black arrow**). Fluorescence immunostaining showed strong granular C3 staining in both mesangium and capillary loops (Fig. 1d). Electron microscopy showed proliferation of mesangial cells, mesangial expansion (Fig. 1e**, star**) and subepithelial immune deposits (Fig. 1f**, black arrow**). She received oral prednisolone 30 mg/day and was gradually tapered between January 2012 and February 2013. She had no family history or history of smoking.

**Fig. 2:**
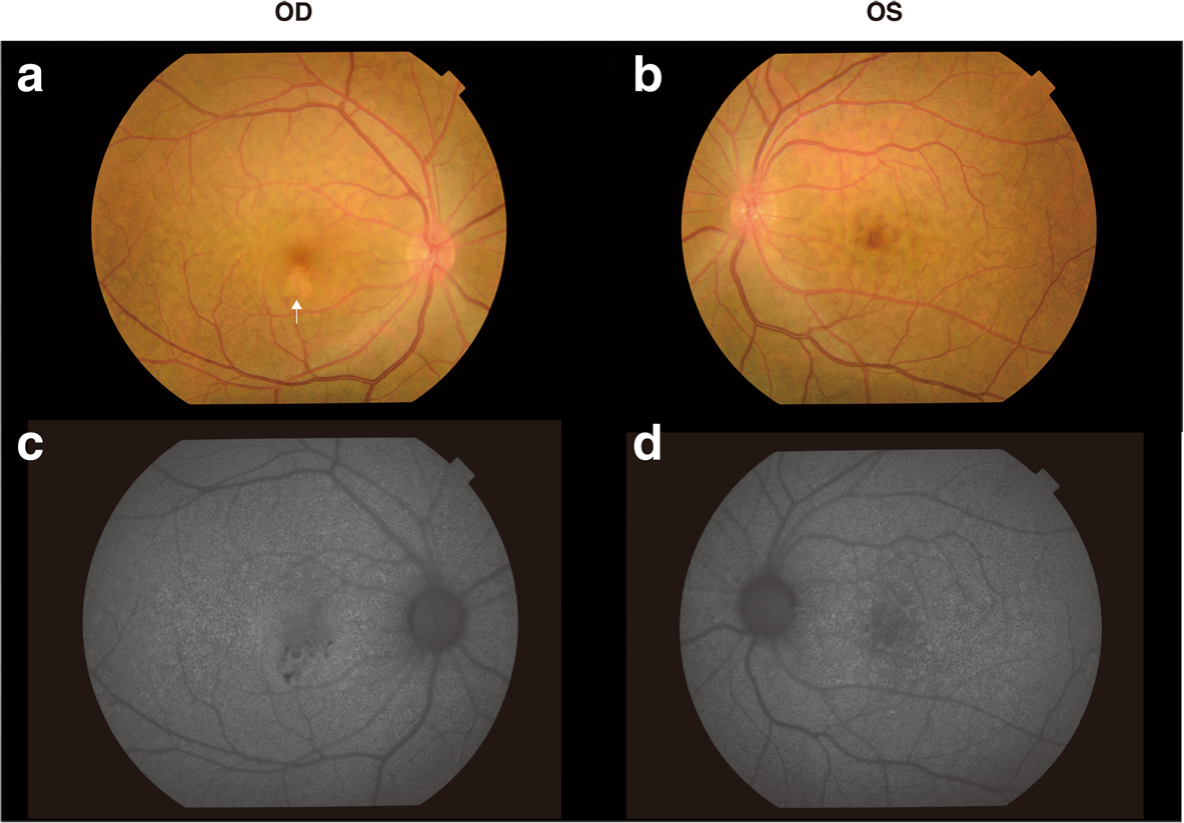
Fundus photograph and autofluorescein angiography. Yellow-white granular patches at the central macula in both eyes (**a** and **b**). Fibrin tissue and a small hemorrhage in the right central fovea (**a**, **white arrow**). Granular hypofluorescences surrounded by a ring-shaped hyperfluorescence in both eyes (**c** and **d**)

**Fig. 3:**
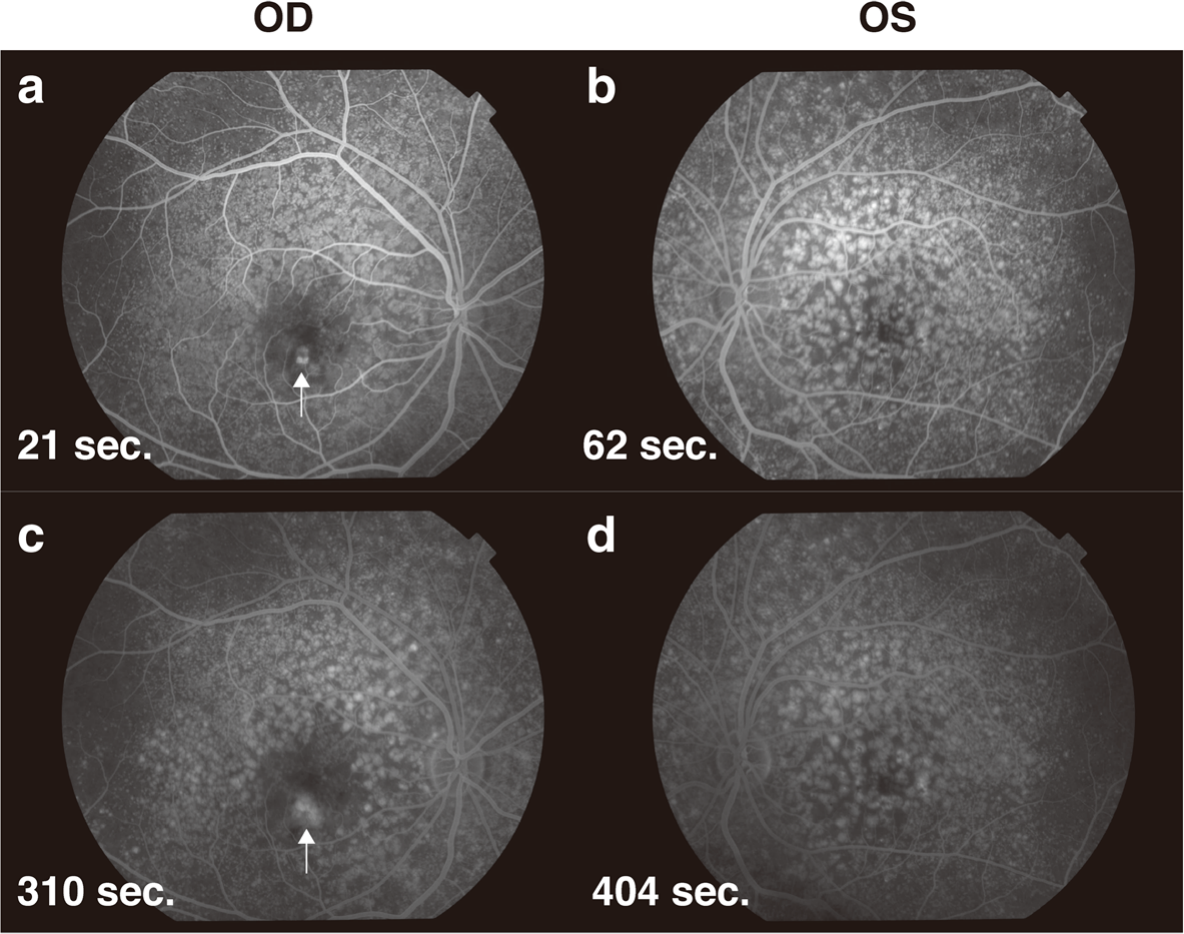
Fundus fluorescein angiography. Numerous granular hyperfluorescence without fluorescence leakage at the site corresponding with the various shaped protrusions of retinal pigment epithelium (RPE) from the early phase of both eyes (**a** and **b**). Hyperfluorescence leakage from choroidal neovascularization of the right eye (**a** and **c**, **white arrow**). No leakage at the areas of protrusions of RPE in the late phase of the left eye (**d**)

**Fig. 4:**
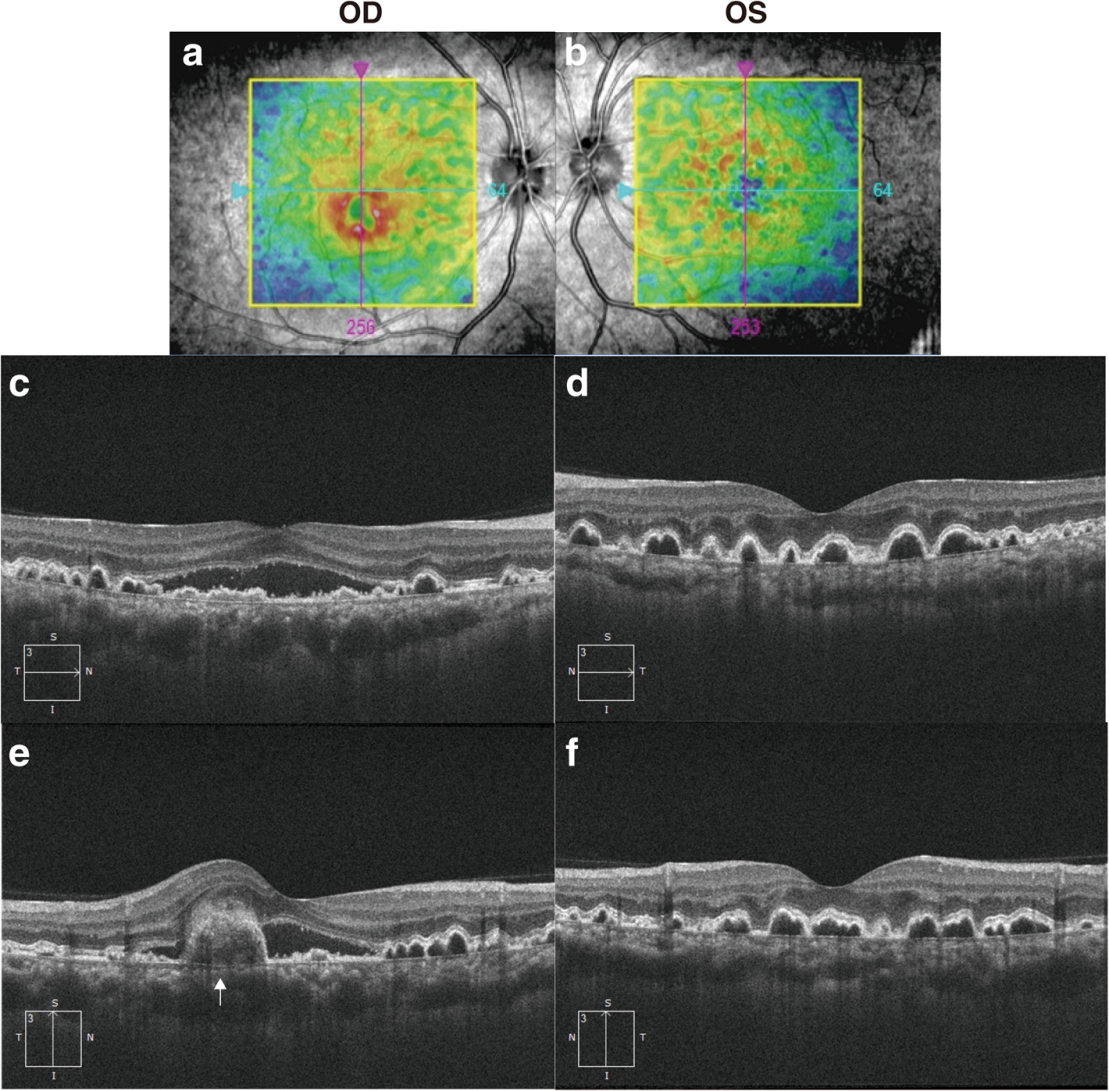
OCT images. The overlay of the line scanning ophthalmoscope retinal image and retinal thickness color map represents the bump of RPE (**a** and **b**). The OCT image showed multiple dome-shaped elevations of RPE in both eyes (**c-f**). CNV with SRD in the right eye demonstrated on vertical OCT image (**e**, white arrow)

In December 2016, her visual acuities were 20/20 OD and 20/16 OS. The intraocular pressures were 15 mmHg OD and 18 mmHg OS. Slit-lamp biomicroscopic exam revealed no abnormal findings in the anterior segment. No inflammatory cells were observed in the anterior chamber and vitreous cavity in both eyes. Fundus exam revealed numerous yellow-white granular patches at the central macula in both eyes (Fig. 2a and b). Fibrin tissue and a small hemorrhage was noted at the lower part of the central fovea of the right eye (Fig. 2a, **white arrow**). Fundus auto fluorescence (TRC-DX50, TOPCON, Fig. 2c and d) showed numerous granular hypofluorescence surrounded by a ring shaped hyperfluorescence in both eyes. Fluorescein angiography showed granular hyperfluorescence at the posterior pole of both eyes without fluorescein leakage in the late phase (Fig. 3a and b). Fluorescein leakage from CNV was noted at the inferior parafovea of the right eye (fig. 3a and c, **white arrow**). There was no leakage at the area of the ring shaped hyperfluorescences in the late phase of the left eye (Fig. 3d). Optical coherence tomography (OCT, CIRRUS HD-OCT model 4000, Carl Zeiss Meditec, Dublin, CA) showed various shaped protrusions of retinal pigment epithelium (RPE) in both eyes (Fig. 4a and b). Apical and basal borders of the RPE showed higher reflection than normal and granular hyperreflective substances were noted at the basal side of the RPE. There was low reflection between the basal side of RPE and Bruch’s membrane at the protruded lesion of RPE. Vertical scan imaging showed CNV and subretinal fluid in the right eye (Fig. 4c**, arrow head**) and fibrin tissue was noted above the CNV. Full field electroretinogram (ERG, TOMEY LE-2000, TOMEY, Nagaya, Japan) showed normal responses in both eyes.

**Fig. 5:**
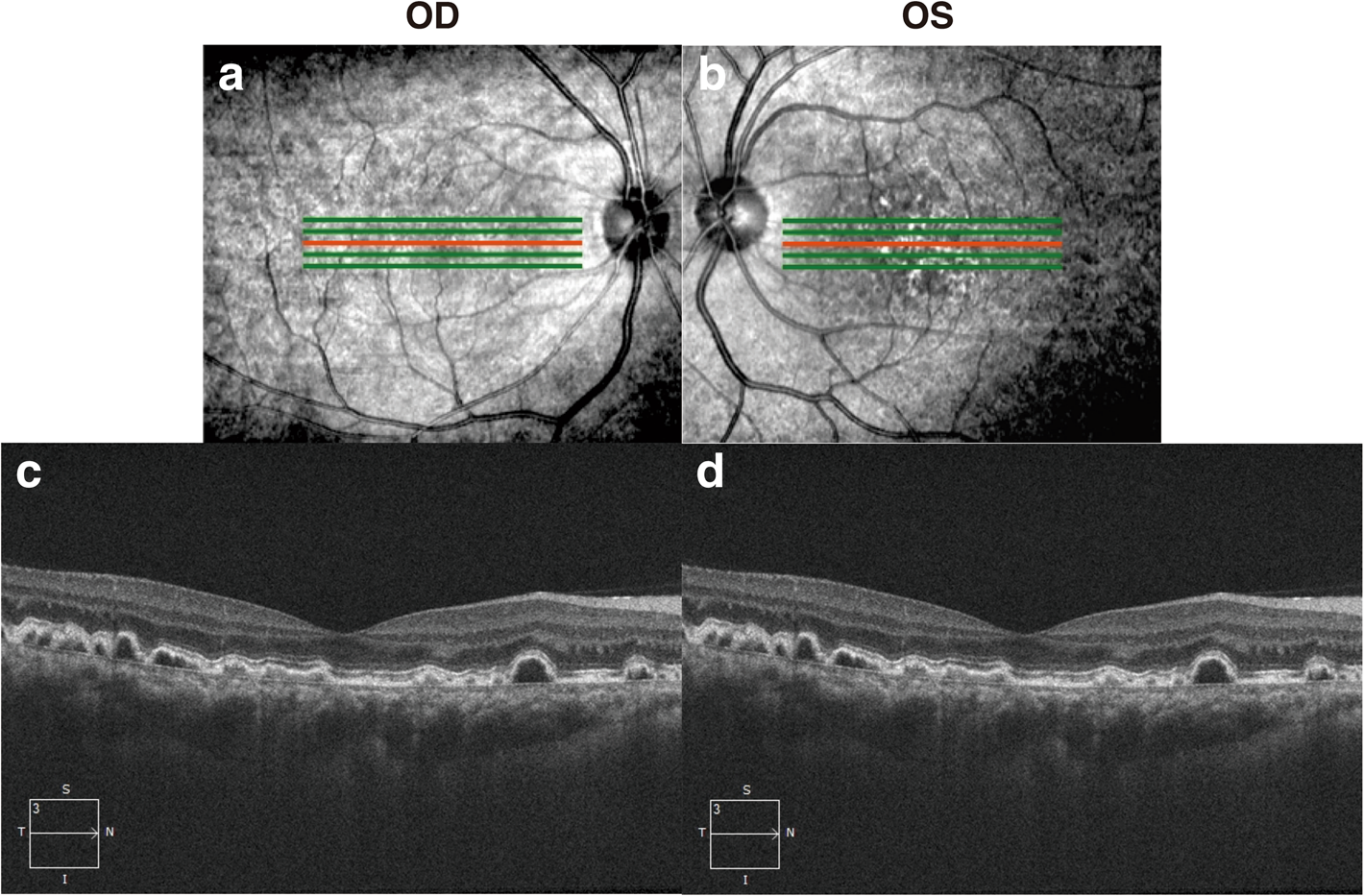
The retinal changes after 1 year. The line scanning ophthalmoscope retinal image noted protrusion of RPE (**a** and **b**). The horizontal OCT images showed the multiple dome-shaped elevations of RPE in both eyes (**c** and **d**). The CNV and SRD in the right eye disappeared (**c**)

Serous retinal detachment (SRD) in the right eye disappeared at 11 months after the initial visit and CNV in the right eye disappeared spontaneously with resolution of subretinal fluid at 12 months after the initial visit. (Fig. 5a and c). However, multiple dome-shaped elevations of RPE remained in both eyes (Fig. 5a-d). At 12 months after the initial visit, visual acuities were 20/16 OD and 20/16 OS and there was no recurrence of CNV with SRD.

## Discussion

MPGN, depending on the subtype, is progressive and has poor prognoses, and may share a common pathogenic mechanism. The presence of drusen-like deposits in MPGN type II is well-known. In 1989, Duvall-Young et al. reported drusen-like deposits in MPGN type II [9, 10] and demonstrated that these deposits shared a similar appearance to the deposits in the kidney on electron microscopy. However, there are few documented cases of MPGN type III with retinal changes. Michielsen et al. observed 17 patients with MPGN type III without fundus changes [11]. However, Dalvin et al. [4] and our case report demonstrated drusenoid deposits in the RPE, suggesting that MPGN type III exhibits various fundus findings.

MPGN has traditionally been considered a group of morphologically classified diseases. Recently, the role of the complement system in MPGN has been recognized and there has been a proposed reclassification of MPGN as an immunoglobulin-mediated disease (via the classical complement pathway) and non-immunoglobulin-mediated disease (via the alternative complement pathway) [4, 12, 13]. Whereas MPGN type II results from disinhibition of the alternative complement pathway, MPGN types I and III results from circulating immune complex deposition that activates the classic complement pathway. MPGN type II is characterized by electron dense deposits of complement components in the glomerular basement membrane and Bruch’s membrane [9]. Our case of MPGN type III showed fundus changes similar to those found in MPGN type II [11] rather than to MPGN type I [8]. This variation of fundus changes ranging from no fundus abnormalities to multiple deposits supports the hypothesis of distinctive forms of glomerulonephritis with distinct etiologies, each varying in duration and disease severity [14].

Generally, the prognosis of visual acuity in MPGN type II is poor due to RPE detachment and CNV occurring in advanced stages [11, 15]. Based on the previous studies [4, 11], the prognosis of visual function in MPGN type III is thought to be better. However, our case of MPGN type III demonstrated CNV with SRD and multiple drusen-like deposits similar to fundus changes in MPGN type II. Those deposits resemble the drusen of age-related macular degeneration because their positions coincided with the drusen between the Bruch’s membrane and RPE in OCT (Fig. 4), although the content of the deposits were unknown. The deposits showed hypoflurescence at the center with surrounding hyperfluorescense in fundus autofluoresence imaging. This appearance was consistent with OCT findings that deposits showed higher reflectivity in the apical and basal sides and low reflectivity inside the deposits. A previous study reported that early laser treatment for an extrafoveal subretinal CNV and anti-VEGF intravitreal injection for subfoveal CNV was effective in treating MPGN type II [16, 17]. The CNV resolved spontaneously in this case similar to previous case report [18], but careful fundus follow-up and a laser treatment or anti-VEGF intravitreal injection may also may help prevent possible vision loss in MPGN type III.

## Conclusions

In summary, we described a case of MPGN type III accompanied by multiple drusen-like deposits and protrusion of RPE associated with CNV and SRD. Retinal lesions due to MPGN may result in visual impairment, and it is very important to devise a logical treatment approach based on MPGN etiology.

## Abbreviations

CNV: Choroidal neovascularization
DDD: Dense deposit disease
HPF: High power field
MPGN: Membranoproliferative glomerulonephritis
OCT: Optical coherent tomography
RPE: Retinal pigment epithelium
SRD: Serous retinal detachment
